# Chemical and biological sensors based on defect-engineered graphene mesh field-effect transistors

**DOI:** 10.1186/s40580-016-0075-9

**Published:** 2016-07-11

**Authors:** Seunghee H. Cho, Sun Sang Kwon, Jaeseok Yi, Won Il Park

**Affiliations:** Division of Materials Science and Engineering, Hanyang University, Seoul, 04763 South Korea

**Keywords:** Graphene, Graphene mesh, Edge-defect, Defect-passivation, Nanosensor, Biosensor

## Abstract

Graphene has been intensively studied for applications to high-performance sensors, but the sensing characteristics of graphene devices have varied from case to case, and the sensing mechanism has not been satisfactorily determined thus far. In this review, we describe recent progress in engineering of the defects in graphene grown by a silica-assisted chemical vapor deposition technique and elucidate the effect of the defects upon the electrical response of graphene sensors. This review provides guidelines for engineering and/or passivating defects to improve sensor performance and reliability.

## Introduction

Graphene, a two-dimensional (2D) zero-gap semiconductor, has drawn great interest as a promising platform for novel electronic, optoelectronic, and energy harvesting systems [1–9]. In particular, application to sensors has been explored because graphene’s one-atomic 2D nature allows its electrical characteristics to be sensitively influenced by the surrounding chemical and biological environment [10–20]. Moreover, graphene has excellent electrical conductivity and mobility [5, 21–23] as well as a low level of 1/f noise [10, 12], which might even enable the real-time electrical detection of single-molecular binding events.

However, the performance of graphene sensors has varied greatly among reported works [12, 24–28]. This variation has been ascribed to the quality of graphene, which is determined by the synthesis and fabrication processes used; yet the relevant mechanisms, and especially the role of defects, have remained poorly understood thus far. For example, Ang et al. reported large Dirac point shifts of graphene field-effect transistors under changes in acidity (i.e., pH response) of 99 mV/pH, which is even higher than the Nernst limit of 59 mV/pH [10] others observed much smaller pH response when the defects in graphene were passivated with hydrophobic fluorobenzene molecules [24]. On the other hand, Tan et al. found a significant enhancement of the pH response in graphene nanoribbon sensors [25]. The sensitivity improvement was attributed to binding of OH^−^ ions to edge defect sites, but the binding characteristic was not thoroughly determined. Nevertheless, existing methods for introducing defects in graphene entail difficulties in controlling the quantity of the defects and/or avoiding contamination from external substances [29]. Due to such unavoidable side effects, the specific influence of defects upon sensing characteristics and sensing mechanism remain largely unclear.

In an effort to address this issue, a new fabrication strategy was developed to directly synthesize graphene mesh structures [30]. This approach allows the engineering of graphene defects and enables further investigation of their effect upon graphene-based sensor characteristics. Sensors based on graphene mesh have shown unprecedented detection characteristics compared to those of normal graphene sensors. For example, in the case of gas sensors based on Pd nanoparticle-decorated graphene mesh (Pd-GM), defects lowered the energy barrier during carrier injection at the Pd/graphene junction, thereby enhancing sensitivity and allowing faster response and recovery [31]. On the other hand, under a physiological environment where the graphene surface was directly exposed to electrolyte solutions, ion species were directly bound to the defect sites by means of strong chemisorption [32]. This reaction was proven to be irreversible and thus would limit its application in multiple-cycle sensor operations.

## Graphene mesh: synthesis and properties

**Fig. 1:**
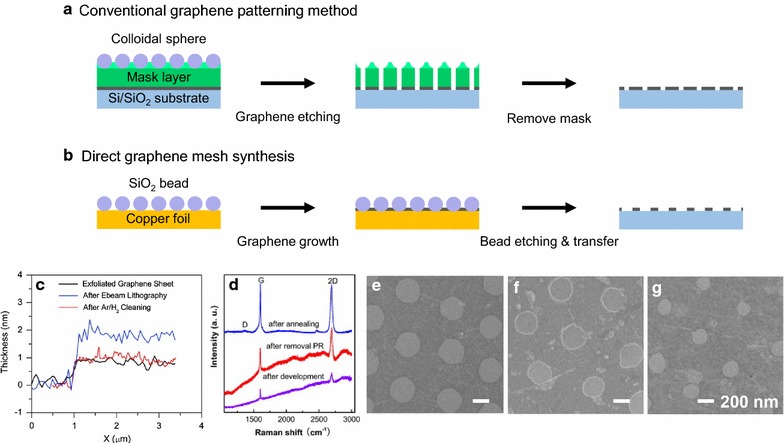
**a**, **b** Schematic illustrations of (**a**) graphene patterning by nanosphere lithography and (**b**) direct synthesis of graphene mesh by a silica-assisted CVD. **c**, **d** Graphene contamination from residual polymers after lithography processes. **e**–**g** SEM images of directly grown graphene meshes having various hole dimensions. **c** Adapted with permission from Ref. [43], © American Chemical Society. **d** Adapted with permission from Ref. [44], © Elsevier. **e**–**g** Reproduced with permission from Ref. [30], © American Chemical Society

Conventional graphene patterning methods have typically been based on top-down processes to achieve well-defined nanoscale patterns. Graphene nanoribbons and graphene nanomeshes have been produced by various methods including unzipping of carbon nanotubes (CNT) [33], e–beam lithography [34–36], block copolymer lithography [37, 38], and nanosphere lithography (Fig. 1a) [39–41]. However, these lithographic techniques inevitably involve contamination by residual polymer and disordered C atoms at the edges caused by the reactive ion bombardment [29, 42–47]. For example, Fan et al. and Dan et al. reported that resists used in photolithography and e-beam lithography caused contamination to graphene, as confirmed by the appearance of D peaks and broadening of characteristic peaks in the Raman spectra of graphene after the lithography process (Fig. 1c, d) [43, 44]. In addition, field-effect transistors (FETs) fabricated with as-patterned graphene showed decreased conductance and large Dirac point shifts owing to unintentional defect doping [43]. Peltekis et al. showed that mild plasma etching can remove such contamination, but with the adverse effect of plasma damage that produces disordered C atoms [46].

**Fig. 2:**
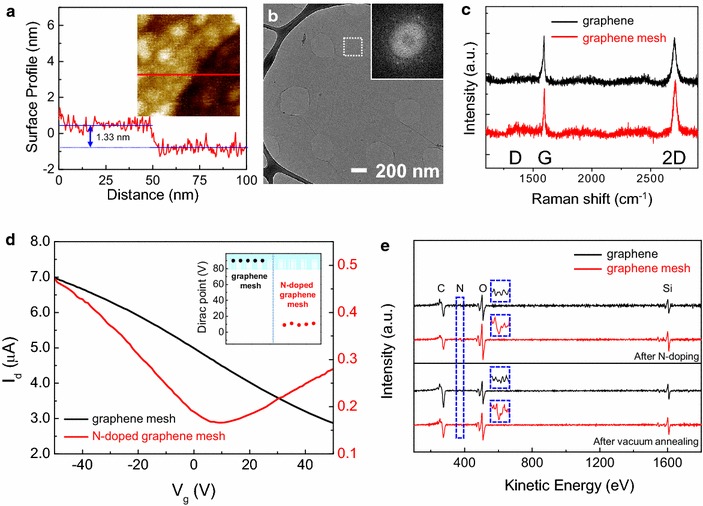
**a** AFM analysis of graphene mesh along the line shown in the *inset*. **b** TEM image and FFT conversion of graphene mesh. **c** Raman spectra of typical graphene and graphene mesh. **d** I_d_–V_g_ curves and Dirac point variations of graphene mesh before and after N doping. **e** AES spectra of N-doped graphene and N-doped graphene mesh (*top*) before and (*bottom*) after vacuum annealing. **a**–**e** Reproduced with permission from Ref. [30], © American Chemical Society

Recently, a new fabrication method for the direct synthesis of graphene mesh has been introduced to minimize the contamination issues associated with graphene patterning (Fig. 1b) [30]. In this method, graphene meshes are synthesized by chemical vapor deposition (CVD) on a metal catalyst, using self-assembled silica spheres as a mask layer to suppress graphene growth. Owing to catalytic hydrogenation, C species are selectively dissociated underneath the silica spheres and thereby producing the mesh structure [48–51]. Graphene meshes were transferred to target substrates using a poly(methylmethacrylate) (PMMA) protecting layer according to a method described elsewhere [5, 30], followed by thermal annealing in vacuum to minimize the contamination from residual polymers. Scanning electron microscopy (SEM) images also show how it is possible to tune the dimensions of the holes in the mesh structure by controlling the size of the silica spheres and/or the depth of silica sinking (Fig. 1e–g). Topographical atomic force microscopy (AFM) analysis of graphene mesh has indicated very abrupt edges, and structural analysis by transmission electron microscopy (TEM) and Raman spectroscopy have confirmed clean and empty holes within graphene of high structural quality (Fig. 2a–c).

Because the graphene meshes are directly synthesized, their edges are less vulnerable to contamination than those produced by lithographic methods. To further examine the characteristics of the edge defects, doping of graphene meshes was explored by high-temperature thermal annealing under NH_3_ atmosphere. In the drain current versus gate voltage (I_d_–V_g_) curves of graphene mesh FETs, the Dirac point was shifted greatly in the negative direction after N doping. During the annealing process, physisorbed molecules such as O species [52, 53] were desorbed and N elements were covalently functionalized at the edges of graphene meshes, moving the Dirac point to the left-hand side (Fig. 2d). Such successful N doping results can be associated with the clean and abrupt nature of the graphene mesh edges, which allows them to be chemically reactive. The stable doping through strong C–N bonds in the N-doped graphene mesh was also confirmed by a distinct N peak in Auger electron spectroscopy (AES) spectra that remained even after additional vacuum annealing (Fig. 2e).

## Pd–graphene mesh hybrid gas sensors

Pd shows high reactivity and resistance change upon exposure to H_2_, even at room temperature [54–57], making it a promising material for H_2_ sensing. However, rigid Pd films undergo structural degradation during reaction with H_2_ [54, 57–60]. To overcome this problem of Pd film sensors, the use of Pd nanoparticles-semiconductor hybrid structures has been proposed such as Pd-GaAs [61], Pd-Si [62], and Pd-CNT [57, 63–65]. In these sensors, the charge carriers generated during Pd hydridation are transported to the semiconductor channels and modulate the resistance. More recently, as an alternative to the existing channel materials, graphene was proposed to take advantage of its 2D nature. In the resulting Pd-graphene (Pd-Gr) sensors, however, charge carrier injection from Pd to the chemically inert graphene surface was limited by the relatively high contact barrier [66, 67]. Accordingly, the presence of defects in the graphene modulates the contact barrier and thus plays an important role in the sensing characteristics.

**Fig. 3:**
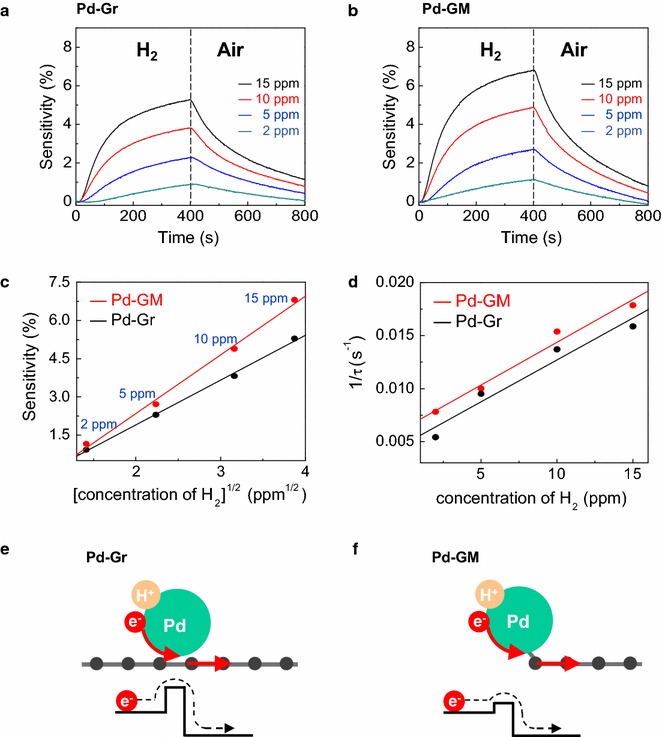
**a**, **b** Sensitivity measurements of Pd-Gr and Pd-GM sensors at different H_2_ gas concentrations. **c** Sensitivities as a function of the square root of H_2_ concentration, and **d** reciprocal response time (1/*τ*) in relation with H_2_ concentration. **e**, **f** Schematic of electron transport mechanism for Pd on graphene and graphene mesh. **a**–**d** Reproduced with permission from Ref. [31], © IOP Publishing

To investigate the influence of graphene defects upon sensing characteristics, H_2_ gas sensors have been fabricated with normal Pd-Gr and with Pd-GM [31]. Sensing characteristics of these Pd-Gr and Pd-GM devices were tested under various concentrations of H_2_ gas, and the continuous changes in resistance were monitored at room temperature. The relative resistance changes were greater for Pd-GM sensors upon exposure to H_2_ gas of concentrations ranging from 2 to 15 ppm (Fig. 3a, b). The device sensitivity (R − R_0_)/R_0_, where R_0_ and R respectively denote the channel resistances before and after exposure to H_2_ gas, improved by 18.2–28.8 % when graphene was replaced with graphene mesh (Fig. 3c). In addition, analysis of response time (*τ*) showed that Pd-GM sensors responded slightly faster than Pd-Gr to H_2_ gas (Fig. 3d). The enhanced sensitivity as well as the faster response of Pd-GM sensors is strongly associated with the existence of energetically active edges along the holes of the graphene mesh [30]. Whereas electron transfer from Pd to pristine graphene occurs mainly across a high energy barrier [66, 67], edges in the graphene mesh provide defect sites that present a lower energy barrier, thereby enabling greater and faster charge carrier transport (Fig. 3e, f).

It is also noteworthy that the response of Pd-GM sensors to H_2_ was reversible; their resistances returned to their initial values when the reactor was purged with air (Fig. 3a, b). This indicates that the Pd hydridation reaction is reversible and also that the edge defects are not directly associated with the reaction with H_2_.

## Graphene mesh pH sensors

**Fig. 4:**
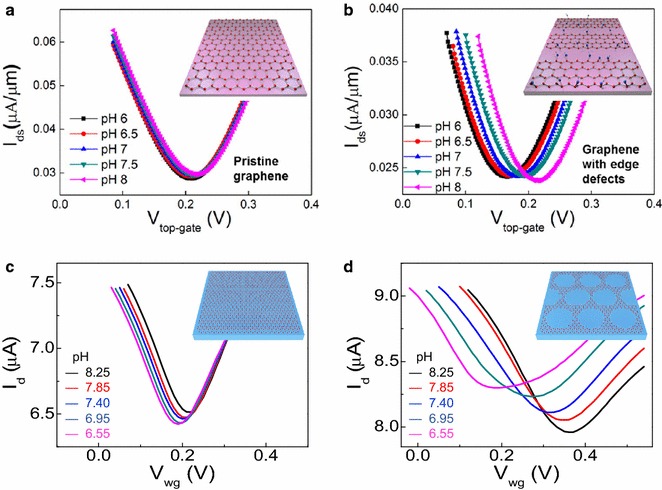
I_d_–V_g_ curves for electrolyte-gated graphene-based FETs in various pH buffer solutions. **a** Pristine graphene, **b** graphene nanoribbons, **c** normal graphene, and **d** graphene mesh. **a**, **b** Adapted with permission from Ref. [25], © American Chemical Society. **c**, **d** Reproduced with permission from Ref. [32], © American Chemical Society

Both physiological and biological environments are characterized by changes in ionic concentration, and thus pH sensing functions are essential in real-time monitoring of biological events. In graphene-based FET sensors, sensing occurs promptly after the adsorption and desorption of chemical and biological species to the graphene surface, causing the so called ‘chemical gating’ effect whereby there is a shift in the Dirac point voltage [68]. In addition to the chemical gating effect, a ‘defect doping’ effect has been recently proposed to suggest the concept of direct charge carrier transfer between adsorbed species and graphene [43, 69, 70]. In the latter case, graphene defects can significantly influence the sensing characteristics by providing sites for strong interactions with ionic species [39, 71–73]. As an example of controllably introducing defects, graphene nanoribbon sensors have been reported to have enhanced pH sensitivities by enabling the direct binding of ions to edge defects (Fig. 4a, b) [25]. Nevertheless, the exact mechanism associated with the binding events and the reversibility of the reaction remains largely unclear.

To investigate the effect of defects in graphene-based FET pH sensors, electrolyte-gated graphene-FET (Gr-FET) and graphene mesh–FET (GM-FET) devices have been tested [32]. Both sensors exhibit negative Dirac point shifts upon decreases in pH. Whereas Gr-FETs have shown sensitivities of ~16.2 mV/pH, those of GM-FETs were significantly higher, at ~89.7 mV/pH (Fig. 4c, d). Typically, the pH sensitivities of GM-FETs were on average ~3 times higher than that of normal Gr-FETs, and also they frequently exceeded the thermodynamically allowed maximum limit (i.e., the Nernst limit) of 59 mV/pH. This result illustrates that the Dirac point shift is not driven solely by the electrostatic gating effect arising from physisorption of H^+^ ions on the Gr surface; rather, this result suggests an additional defect doping effect whereby unsaturated C atoms at the graphene mesh edges (i.e., defect sites) provide binding sites for the H^+^ ions, thereby further increasing the Dirac point shifts in the GM-FETs.

**Fig. 5:**
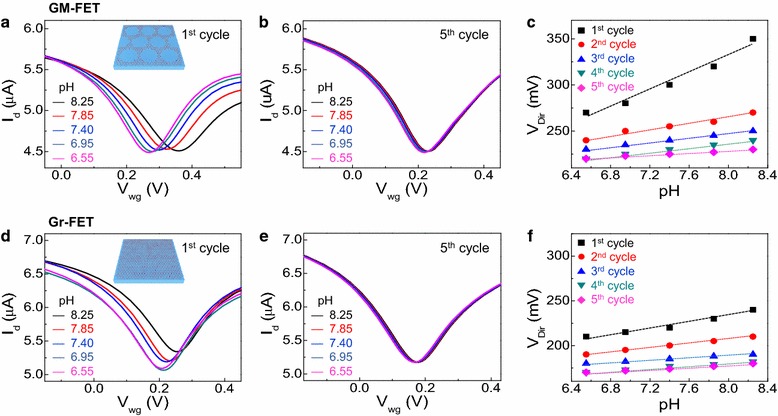
Cyclic I_d_–V_g_ characteristics of (**a**–**c**) GM-FETs and (**d**–**f**) Gr-FETs: **a**, **b**, **d**, **e** I_d_–V_g_ characteristic curves during the (**a**, **d**) first cycle and (**b**, **e**) fifth cycle; (**c**, **f**) corresponding plots of Dirac point voltages during five cycles. **a**–**f** Reproduced with permission from Ref. [32], © American Chemical Society

Further analysis has been carried out to examine the influence of defect–H^+^ ion binding upon the cycling behaviors of graphene-based FET sensors. During cyclic tests in which GM-FET sensors were repeatedly exposed to conditions of various pH from 8.25 to 6.55, large Dirac point shifts of 90 mV were observed in the first cycle (Fig. 5a). Although the Dirac point did not recover its original state when the pH was restored to its initial value during the first cycle, the Dirac point shifts decreased gradually with cycling, and after five cycles converged to ~10 mV per cycle (Fig. 5b). As a result, the I_d_–V_g_ characteristics in response to pH became reversible, with corresponding sensitivities of ~7 mV/pH (Fig. 5c).

Gr-FETs have shown results consistent with those of GM-FETs: there was an irreversible portion of the response that gradually decreased with cycling, and only a reversible component remained after five cycles (Fig. 5d–f). The magnitude of the irreversible component of the Dirac point shift in Gr-FETs (~30 mV) was considerably smaller than that in GM-FETs (~90 mV), whereas the remaining reversible component was similar for both FETs (~7.0 mV/pH). This reflects the fact that both intrinsic and extrinsic defects provided interaction sites for H^+^ ions, whereas the increased number of defects at the mesh edges enhanced the pH response of the GM-FETs during the initial cycles. Furthermore, the irreversible response is believed to be caused by the direct adsorption of ions onto defects, which presumably involved strong chemisorption. It has been reported that H^+^ ions were attached so strongly to the edge defects and have only been detached after high temperature annealing [32]. Such strong interactions were rarely reversed, and thus led to passivation of the defects upon repeated exposure to acidic solutions. This passivation eliminated the irreversible component of the Dirac point shift, thereby enabling stable and reversible pH sensing.

## Conclusions

In this review, we investigated the defects of graphene meshes and their influence upon various sensor applications. Direct growth of graphene mesh by silica-assisted CVD is an excellent way to produce graphene with contamination-free defect sites. When these defects are introduced to Pd-GM gas sensors, the lowered energy barrier at the junctions between Pd nanoparticles and edge defects enhance indirect charge carrier injection into the graphene channel. As a result, the sensitivity and response time of Pd-GM gas sensors have been greatly improved over those of other graphene-based sensors. In contrast, graphene mesh pH sensors respond directly to H^+^ ions at edge defects, which results in increased sensitivities that sometimes exceeded the Nernst limit. Unfortunately, such direct interactions involve irreversible covalent bonding, and therefore are not preferred in multiple-cycle sensor operations. For graphene mesh pH sensors, this issue has been solved through a simple process of passivating the edge sites. These results suggest how graphene mesh edge defects can improve sensors’ sensitivity and response time and also enable stable multiple-cycle operation through indirect carrier injection. As an example, biological sensors can be constructed by including graphene mesh for enhanced sensing characteristics, while improving stability by attaching appropriate receptors to the graphene edge sites to inhibit direct carrier injection.
